# Local electron-electron interaction strength in ferromagnetic nickel determined by spin-polarized positron annihilation

**DOI:** 10.1038/srep20898

**Published:** 2016-02-16

**Authors:** Hubert Ceeh, Josef Andreass Weber, Peter Böni, Michael Leitner, Diana Benea, Liviu Chioncel, Hubert Ebert, Jan Minár, Dieter Vollhardt, Christoph Hugenschmidt

**Affiliations:** 1 Technische Universität München, Lehrstuhl E21, James-Franck Straße, 85748 Garching, Germany; 2 Heinz Maier-Leibnitz Zentrum (MLZ), Technische Universität München, Lichtenbergstraße 1, 85748 Garching, Germany; 3 Chemistry Department, University Munich, Butenandstraße 5-13, 81377 München, Germany; 4 Faculty of Physics, Babes-Bolyai University, Kogălniceanustr 1, 400084 Cluj-Napoca, Romania; 5 Theoretical Physics III, Center for Electronic Correlations and Magnetism, Institute of Physics, University of Augsburg, 86135 Augsburg, Germany; 6 Augsburg Center for Innovative Technologies, University of Augsburg, 86135 Augsburg, Germany; 7 New Technologies - Research Center, University of West Bohemia, Univerzitni 8, 306 14 Pilsen, Czech Republic

## Abstract

We employ a positron annihilation technique, the spin-polarized two-dimensional angular correlation of annihilation radiation (2D-ACAR), to measure the spin-difference spectra of ferromagnetic nickel. The experimental data are compared with the theoretical results obtained within a combination of the local spin density approximation (LSDA) and the many-body dynamical mean-field theory (DMFT). We find that the self-energy defining the electronic correlations in Ni leads to anisotropic contributions to the momentum distribution. By direct comparison of the theoretical and experimental results we determine the strength of the local electronic interaction *U* in ferromagnetic Ni as 2.0 ± 0.1 eV.

The physical properties of ferromagnetic Ni are strongly influenced by correlation effects originating from the Coulomb interaction between electrons in the partially filled, relatively narrow 3*d* band^1^. Due to these correlations density functional theory (DFT)^2^,^3^,^4^ alone cannot explain various experimentally observed features of the electronic structure of Ni. To obtain a realistic description of the electronically correlated materials, parameters such as the *local* Coulomb repulsion *U* need to be employed. The “Hubbard” parameter *U* was originally introduced for single-band models^5^,^6^ and is defined as the Coulomb energy required to place two electrons on the same site: 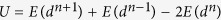. Here 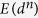 represents the total energy of a system for which *n* electrons fill a given *d*-shell on a given atom. In multi-band systems *U* takes the form of an interaction matrix.

The Hubbard model and related lattice models are able to explain important general features of correlated electrons, but they cannot describe the physics of real materials in detail. Namely, for an approach to be realistic it has to take into account the lattice and the explicit electronic structure of the systems. Here significant progress was made through the combination of DFT in the local density approximation (LDA) with dynamical mean field theory (DMFT)^7^,^8^,^9^,^10^ which is generally referred to as the LDA + DMFT method^10^,^11^. In the case of magnetic states the local *spin* density approximation (LSDA) rather than the LDA needs to be employed. In the L(S)DA + DMFT scheme the L(S)DA provides the *ab initio* material dependent input (orbitals and hoping parameters), while the DMFT solves the many-body problem for the local interactions. Therefore the LSDA + DMFT approach is able to compute, and even predict, properties of correlated materials. Considerable effort has been undertaken to construct systematic extensions beyond LSDA + DMFT in which ab-initio and interaction parameters are computed on the same footing in a self-consistent way. However, there remain many open questions due to the considerable algorithmical and numerical difficulties of the probelm (see ref. 10. and references therein).

Theoretical results obtained with LSDA + DMFT can be compared with experimental data obtained, for example, by photoemission spectroscopy (PES). In particular, this technique measures spectral functions, i.e., the imaginary part of the one-particle Green function, and thus determines correlation induced shifts of the spectral weight. This allows one to estimate the local Hubbard interaction *U* of a material, say, Ni. Indeed, most experimental investigations on the electronic structure of Ni rely on PES^12^,^13^. Braun *et al.*^14^ demonstrated the importance of local correlations in Ni by exploiting the magnetic circular dichroism in bulk sensitive soft X-ray PES measurements. One of the dominant correlation effects observed in the PES data for Ni is the satellite peak situated at 6 eV below the Fermi level^15^,^16^. This feature is not captured by LSDA, but is well explained by LSDA + DMFT^17^. LSDA + DMFT also reproduces the correct width of the occupied 3*d* bands and the exchange splitting^17^.

In this paper we discuss an alternative experimental technique to determine the local Coulomb parameter *U*, involving *positrons*. In contrast to photoelectron spectroscopy the theoretical analysis of positron spectroscopy does not suffer from complications due to external effects such as surfaces. We show that by combining experimental results of the spin-polarized two-dimensional angular correlation of annihilation radiation (2D-ACAR) with LSDA + DMFT computations including a careful and realistic treatment of the positron probe effects, it is possible to determine the strength of the electronic interactions in Ni quite unambiguously.

## Results

**Figure 1:**
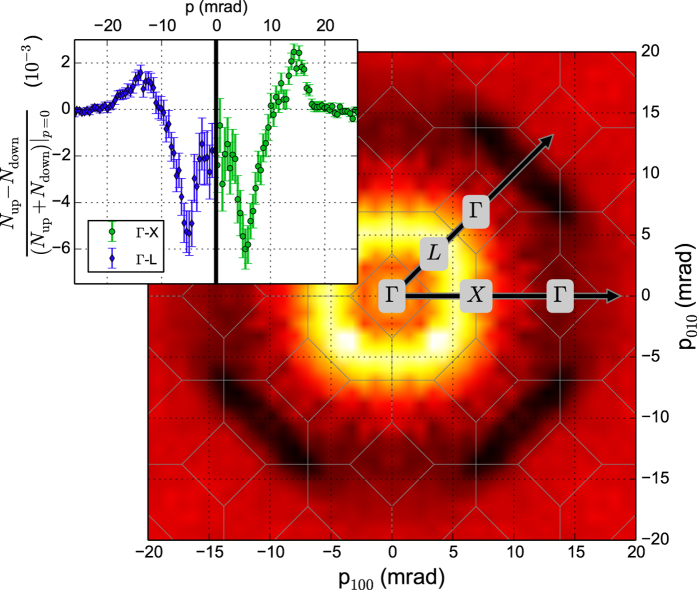
2D-ACAR difference spectra Δ*N*(*P*_*x*_, *p*_*y*_) obtained upon reversal of the magnetic field, with the integration along the [001] direction, *p*_*x*_ = [100] and *p*_*y*_ = [010]. The inset illustrates the anisotropy of the difference spectra between two directions in momentum space.

**Figure 2:**
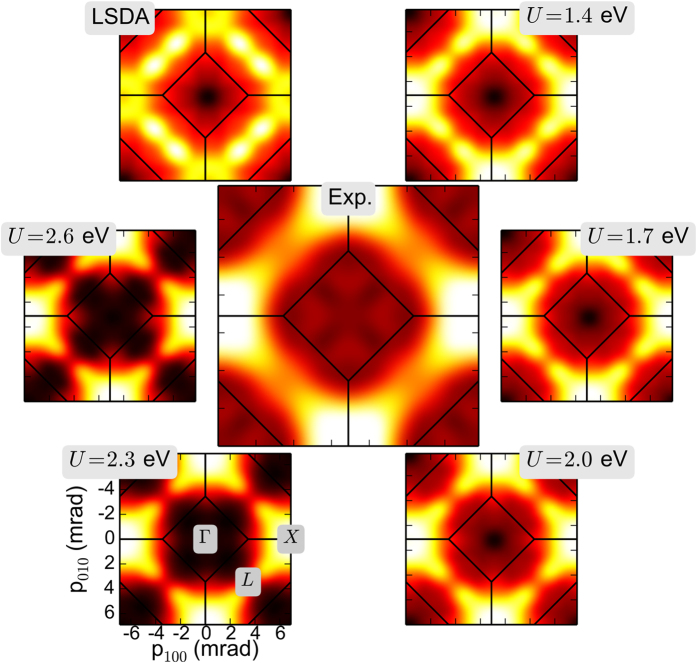
Experimental magnetic LCW spectrum (*center*) compared to theoretical spectra computed for different values of the local Coulomb parameter (LSDA corresponds to *U* = 0 eV in the range from 1.4 to 2.6 eV; see text.

In angular correlation measurements the quantity of interest is the probability per unit time and per unit volume in momentum space for the annihilating positron to produce two gamma quanta with a total momentum **p**, the so-called 2D-ACAR. If we assume for simplicity that the thermalized positron (with zero momentum at zero temeprature) has no correlations with the electrons then the 2D-ACAR gives directly the electron momentum probabilities. It is well known that both the shape and the structure of the 2D-ACAR probability distribution, 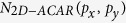, are determined by the nature of wave functions of the electron and positron as well as by the topology of the Fermi surface. To obtain the latter we applied the Lock-Crisp-West (LCW) back-folding procedure both on the measured and the computed data. Since Ni is a ferromagnetic metal, spin-polarized 2D-ACAR measurements have been carried out. The spin-polarized 2D-ACAR measures the difference of the two spectra 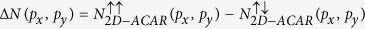 and is related to the spin-dependent momentum density of the material^18^. 
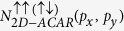
 represents the experimental 2D-ACAR spectrum for the magnetization aligned parallel 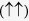 or anti-parallel 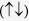 to the positron polarisation. The results are seen in Fig. 1. In each spectrum 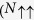 and 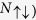 more than 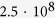 counts were collected, and the data were corrected for the momentum sampling function. Before subtraction, the spectra 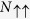 and 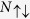 were normalized to an equal amount of counts. A renormalization due to 3*γ* decay was omitted since the corresponding correction in the case of Ni is negligible compared to the statistical noise^18^. The expected 4-fold symmetry is clearly observed and the anisotropy is found to be in good agreement with the study of Genould *et al.*^19^. The inset in Fig. 1 shows that the anisotropy of the magnetic signal, 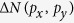, is more pronounced along the Γ – X – Γ direction than along the Γ – L – Γ direction. This is also in accordance with the calculations of Nagoa *et al.*^20^. The comparison between the experimental and theoretical spin-polarized 2D-ACAR data is presented in Fig. 2. Both spectra have been back-folded using the LCW-procedure. The central plot represents the measured spectra, and is surrounded by theoretical results obtained from LSDA + DMFT performed for several values of *U*. Our results show significant electronic correlations effects. It is clearly seen that with increasing *U* a gap opens at the L-points of the Brillouin zone. This gap is associated with the necks in the Fermi surface of Ni. This implies that LSDA underestimates the density at the X-point, while the density near the L-point is overestimated. In the LSDA + DMFT calculation the highest density is found at the X-point, similar to the experiment. However, the structure connecting the X and L-points is less pronounced in the experimental data than in the LSDA + DMFT results. Apparently the local interaction (see e.g.^14^) pushes the *d*-bands below the Fermi energy, whereby the X_2_ hole pocket obtained in LSDA disappears. This is a general feature of electronic correlations in Ni^21^ and also greatly changes the calculated anomalous Hall effect of Ni^22^,^23^. Therefore, our experiment is able to identify the electronic correlations as the origin of anomalous Hall resistivity in bulk Ni.

**Figure 3:**
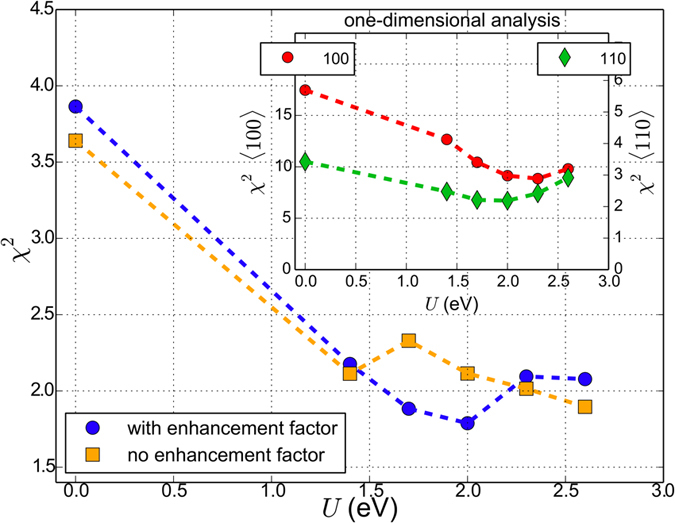
Least square fit analysis (*χ*^2^) between LSDA + DMFT calculations and experimental data as a function of the Hubbard *U* for the 2D data. Higher *U* values correspond to stronger electron-electron correlations. A pronounced minimum of 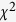 is found for 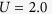
eV. The inset shows the results for the 1D data. (The dotted lines act as a guide to the eye).

In order to derive the value of the local Coulomb interaction parameter *U* from our experiment we performed a least square fit 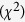 analysis of the measured data with the LSDA + DMFT calculations (Fig. 2). To assess the importance of the electron-positron correlation, two sets of LSDA + DMFT computations have been performed, one without and one with the electron-positron interaction. The results for 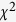 without the electron-positron interaction (“independent particle model” (IPM)) are shown in Fig. 3 by a dashed yellow line. However, the electron-positron attraction should not be neglected since it leads to an increase of electron density at the positron, which is refered to as “enhancement” since the total annihilation rate strongly increases. Indeed, the inclusion of the electron-positron interaction by the so-called enhancement factor (a multiplicative factor in the product of the electron and positron wave functions) changes 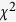 substantially: now a clear minimum in 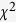 is found (dashed blue line). A more detailed discussion of the enhancement factor is presented in the following section. The minimum in 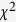 is found at 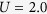 eV. The 2D-ACAR spectra can be projected along different directions in mometum space as shown in the inset of Fig. 3. The 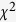 curves of the 1D data exhibit minima for the value of 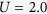 eV for the Hubbard interaction similar to the 2D-ACAR spectra. The loss of information in the doubly integrated 1D data is indicated by a larger 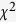 value. We also note that the effects of electron-electron correlations within the LSDA + DMFT on the electron density are anisotropic and therefore go beyond an isotropic Lam-Platzman^24^ correction of the LSDA data. Interpolating the data in Fig. 3 with higher order polynomials allows us to estimate the systematic error in the position of the absolute minimum as ±0.1 eV.

## Discussion

There exist a number of experimental studies on the numerical value of the local Hubbard interaction *U* in Ni. Specifically, J. Braun *et al.* (ref. 14) as well as J. Sánchez-Barriga *et al.* (ref. 15) present results on photoelectron emission studies. While ref. 14 reports on soft x-ray ARPES on bulk Ni, ref. 15 discusses spin resolved ultra-violet ARPES obtained on Ni films grown epitaxially on a tungsten substrate. In an approach that is conceptually similar to ours, they model the experimental energy distribution curves in a LSDA + DMFT framework using a one step model and conclude that *U* in the range of 2.5 to 2.8 eV gives an optimal fit. A somewhat smaller value for *U* is reported in the study of the anomalous Hall effect in Ni by Ködderitzsch *et al.*, yielding a value for the Hubbard interaction parameter of 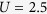 eV^23^. Modelling experimental Compton scattering profiles^25^ by LSDA + DMFT^26^,^27^ gives yet smaller values of 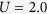 to 2.3 eV, similar to the value of 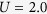 eV as reported here.

At first sight, the variation in the values of *U* determined by the various experimental methods seems displeasing. However, we want to point out the aspect of the electronic states sampled by the methods, specifically with respect to the electron binding energies: In spin-polarized 2D-ACAR electrons close to the Fermi energy contribute most to the measured two-photon-momentum-distributions due to the sampling by the positron (wave-function and correlation effects). In magnetic Compton scattering all electrons contribute equally to the measured Compton profiles as the scattering cross section is virtually independent of the binding energy. Furthermore, in the anomalous Hall effect the electrons in the region with a high density-of-states contribute the most, whereas in photoemission spectroscopy electrons with even higher binding energies (up to −10 eV) are considered.

Therefore, we conclude that while the theoretical modelling of the various methods assumes a single value of *U*, in reality different electronic states would correspond to different values, which are sampled by the diverse experimental methods. In addition, one has to bear in mind that while anomalous Hall effect measurements similarly to Compton or positron-annihilation experiments are bulk sensitive techniques, photo-electron spectroscopy is fairly surface sensitive. In a first approximation it is therefore expected to find a larger value of *U* due to a reduced number of nearest neighbours that screen the value of *U* at the surface.

Furthermore, we also take the opportunity to discuss the electron-positron correlation effects in Ni. A fundamental question in positron annihilation in solids is how the electron-positron interaction modifies the electronic structure of the medium which is being probed. The electron-positron attraction leads to an increase of electron density at the positron, which manifests itself in the annihilation characteristics. This effect is called “enhancement” and is qualitatively well understood: the total annihilation rate is strongly increased. However, apart from the short-range screening, the electronic states and the mean density remain almost unchanged. Therefore, the 2D-ACAR shows only relatively weak differences compared to the independent particle model (IPM). In the case of alkali metals the enhancement effect is included by multiplying the 2D-ACAR spectra computed in the independent particle model with an isotropic enhancement factor^28^,^29^, the so-called Kahana factor. This approach was generalized to an energy dependent form^30^, and was later extended to an orbital dependence^31^. It was formulated within DFT^32^,^33^,^34^,^35^,^36^,^37^,^38^,^39^,^40^,^41^,^42^ and therefore maintains its static mean-field character. Different parametrizations of the enhancement functional have been proposed in the literature^32^,^39^,^40^,^43^,^44^,^45^,^46^ and applied in the case of Ni^31^,^47^.

Biasini *et al.*^48^,^49^. proposed that probe effects associated with electron-positron interaction, such as positron wave function effects, can be partially avoided by applying magnetic 2D-ACAR. We have tested this conjecture, by computing the 2D-ACAR spectra in the presence and absence, respectively, of the positron, with and without electronic and electron-positron correlations. The electron-positron interaction was included in the form of an effective one-particle potential as formulated in DFT by Boronski and Nieminen^32^.

**Figure 4:**
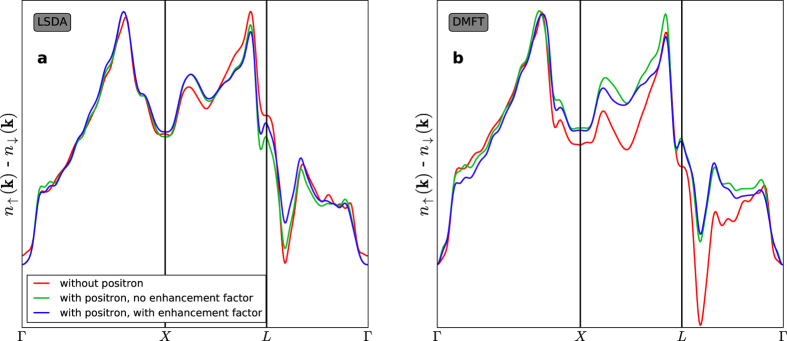
Cuts through the LCW-calculated spin-contrast along major symmetry points. The effect of the positron wave function and the combined effects of the positron wave function and the electron-positron correlations are compared with the pure electron density for calculations performed within the LSDA (**a**) and LSDA + DMFT (**b**) framework.

We analyzed our results by taking several cuts along the symmetry directions in the Brillouin zone. Since the positron affects the individual spin channels differently we plot in Fig. 4 the spin-contrast, 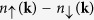, which was computed within LSDA and LSDA + DMFT, respectively. Within LSDA the spin difference is found to be essentially independent of the presence of the positron. In particular, it remains essentially the same along the symmetry lines in the Brillouin zone. Once electronic correlations are included by DMFT a clear difference in the results for the spin-contrast in the presence and absence, respectively, of the positron is found. The results show that the conjecture of Biasini *et al.*^48^,^49^ regarding the cancelation of probe effects in spin-polarized 2D-ACAR is not valid beyond LSDA, i.e., when many-body effects are included. The cancelation appears to be a consequence of the form of the electron-electron and electron-positron correlation functionals in the LSDA, which have a very similar mean-field structure.

## Methods

### Spin-polarized 2D-ACAR.

For a detailed description of the basic 2D-ACAR technique we refer to refs 50, 51, 52.

The spin-polarized variant of 2D-ACAR relies on two aspects: the non-zero net polarisation of positrons from a radioactive source, which was determined as 31(4)% in a separate experiment, and the fact that positron annihilation occurs mainly when the spins of positron and electron are aligned anti-parallel. Spin-polarized 2D-ACAR is one of the few experimental methods that can probe the momentum distribution of the electrons in the bulk with respect to the spin direction and even at elevated temperatures. It was successfully applied to elemental Ni^19^,^53^,^54^ and other materials^55^,^56^,^57^,^58^.

**Figure 5:**
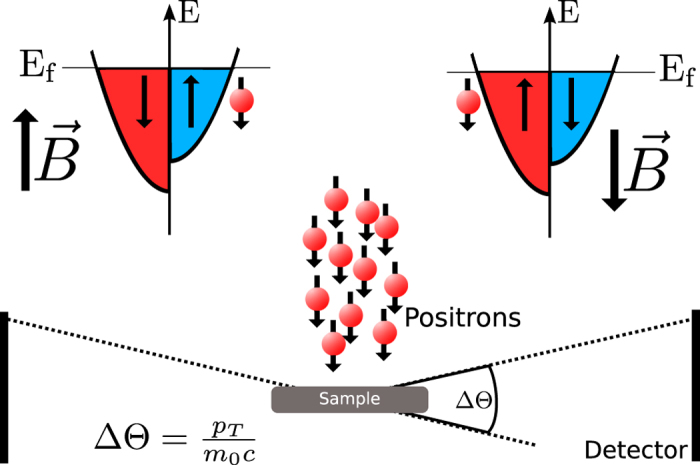
Schematic picture of spin-polarized 2D-ACAR. In electron-positron annihilation the singlet configuration is preferred for majority or minority spin electrons if the magnetization of the sample is parallel or anti-parallel to the emission direction of the positrons.

We measured spin-resolved 2D-ACAR in magnetic fields up to 1.1 T at room temperature. The field was applied parallel and anti-parallel, respectively, relative to the crystallographic 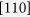 orientation of the sample which coincides with the emission direction of the positrons (i.e. the polarisation direction of the positron beam) as well as the magnetic easy-axis of Ni. When an external magnetic field is applied parallel or anti-parallel to the emission direction the positrons will annihilate predominantly with electrons from the majority or the minority spin directions, respectively (see Fig. 5).

### LSDA+DMFT

The theoretical analysis of the 2D-ACAR spectra requires the knowledge of the two-particle electron-positron Green function, describing the probability amplitude for an electron and a positron propagating between two different space-time points. This two particle many-body problem is factorized into the many-body electronic and the one-body positronic part. The DFT can be generalized to electron-positron systems by including the positron density, in the form of the 2-component DFT^32^,^59^. The electron-positron correlations are taken into account by a multiplicative factor, the so-called enhancement factor, briefly discussed above. Within DFT the enhancement factor is treated as a functional of the electron density in the local density approximation^59^.

Electronic structure calculations were performed with the spin-polarized relativistic Korringa-Kohn-Rostoker (SPR-KKR) method^60^. For LSDA computations the exchange-correlation potentials parametrized by Vosko, Wilk and Nusair^61^ were used with a lattice parameter of 3.52 Å. To include the electronic correlations, a charge and self-energy self-consistent LSDA + DMFT scheme was employed, which is based on the KKR approach^62^ and where the impurity problem is solved with a spin-polarized *T*-matrix fluctuation exchange method^63^,^64^. This impurity solver is fully rotationally invariant even in the multi-orbital version and is reliable when the interaction *U* is smaller than the bandwidth, a condition which is fulfilled in the case of Ni. In this LSDA + DMFT framework the electron-positron momentum density 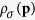 is computed directly from the two-particle Green function in the momentum representation. The neglect of electron-positron correlations corresponds to the factorization of the two-particle Green function in real space. In the numerical implementation the position-space integrals for the “auxiliary” Green function 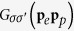 obtained within LSDA or LSDA + DMFT, respectively, are performed as integrals over unit cells:


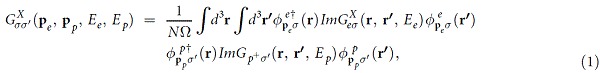


where *X* = LSDA or LSDA + DMFT, and 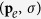, and 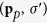 are the momenta and spin of electron and positron, respectively. Here 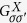 is computed for each energy point on the complex energy contour, providing the electron-positron momentum density:


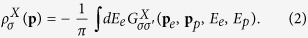


Integration over positron energies 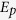 is not required, since only the ground state is considered, and 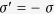 at the annihilation. The momentum carried off by the photons is equal to that of the two particles up to a reciprocal lattice vector, reflecting the fact that the annihilation takes place in a crystal. Hence an electron with wave vector *k* contributes to 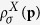 not only at 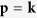 (normal process) but also at 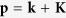, with **K** a vector of the reciprocal lattice (Umklapp process). The experimental spin-difference spectra 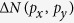 can be compared with the computed difference in the integrated momentum densities of Eq. 2:


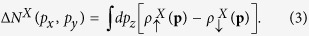


In a perfect bulk material the 2D-ACAR distributions are rather anisotropic, reflecting the fact that certain valence bands in certain directions in the Brillouin zone do not contribute to the momentum density of annihilating electron-positron pairs. One can define the spin-difference spectra: 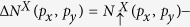

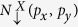
 which was compared with the corresponding experimentally measured spectra 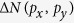 taken in the presence of the magnetic field. Here the annihilation probability for the triplet electron-positron pair is neglected since it is of order 10^-3^ smaller than that of the singlet annihilation.

In order to assess the impact of the presence of the positron, the electronic momentum density was computed from the Green function in the momentum representation, as used in the calculations of the Compton profiles:


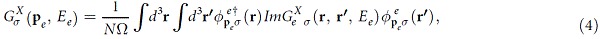


which formally corresponds to Eq. 1 if the positronic Green function is removed. The spin projected momentum density is obtained according to the formula:


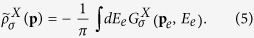


### LCW backfolding

Our analysis concerns the two-dimensional projections of the electronic momentum densities with the integration direction chosen along [100]. The LCW folding procedure^65^ is used for the momentum densities:


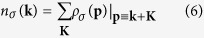


with **K** the reciprocal lattice vector. In the presence of the positron 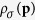 is replaced by 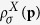 (Eq. 2), while in the absence of the positron 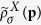 (Eq. 5) takes the place of 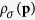 in Eq. 6.

The LCW procedure is exact when applied to the electronic momentum density. In the presence of the positron the back-folding procedure is only exact if the positron wave function is a plane wave. If the positron wave function varies in **k**, completely filled bands also give rise to variations in 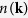. However, the amplitude of the positron wave function varies slowly within the Brillouin zone and therefore the variations in 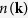 are also expected to be smooth^66^,^67^.

### Sample preparation and characterisation

The Ni crystal (purity 99.99%), with dimensions of 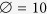 mm × 1 mm, was obtained from MaTecK GmbH. The top surface of the sample is orientated along [110] with an accuracy of ±0.1° and was polished to a surface roughness <50 nm. Positron lifetime measurements prior to the 2D-ACAR measurements revealed only a single lifetime of 113 ps in agreement with the bulk value of Nickel. Hence, it can be safely assumed that the sample is defect-free. Prior to mounting the sample in the spectrometer the orientation of the [100] integration direction was determined by Laue diffraction to ±1°.

## Additional Information

**How to cite this article**: Ceeh, H. *et al.* Local electron-electron interaction strength in ferromagnetic nickel determined by spin-polarized positron annihilation. *Sci. Rep.* **6**, 20898; doi: 10.1038/srep20898 (2016).
