# Erratum: Local electron-electron interaction strength in ferromagnetic nickel determined by spin-polarized positron annihilation

**DOI:** 10.1038/srep23851

**Published:** 2016-04-13

**Authors:** Hubert Ceeh, Josef Andreas Weber, Peter Böni, Michael Leitner, Diana Benea, Liviu Chioncel, Hubert Ebert, Jan Minár, Dieter Vollhardt, Christoph Hugenschmidt

Scientific Reports 6: Article number: 2089810.1038/srep20898; published online: 02162016; updated: 04132016

The original version of this Article contained a typographical error in the spelling of the author Josef Andreas Weber, which was incorrectly given as Josef Andreass Weber. This has now been corrected in the PDF and HTML versions of the Article.

